# Combined rTMS treatment targeting the Anterior Cingulate and the Temporal Cortex for the Treatment of Chronic Tinnitus

**DOI:** 10.1038/srep18028

**Published:** 2015-12-15

**Authors:** Peter M. Kreuzer, Astrid Lehner, Winfried Schlee, Veronika Vielsmeier, Martin Schecklmann, Timm B. Poeppl, Michael Landgrebe, Rainer Rupprecht, Berthold Langguth

**Affiliations:** 1 Department of Psychiatry and Psychotherapy, University of Regensburg, Germany; 2 Department of Psychiatry, Psychosomatics and Psychotherapy, kbo-Lech-Mangfall-Klinik Agatharied, Germany; 3 Department of Otorhinolaryngology, University of Regensburg, Regensburg, Germany; 4 Interdisciplinary Tinnitus Center of the University of Regensburg, Regensburg, Germany

## Abstract

Repetitive transcranial magnetic stimulation (rTMS) has been proposed as a tinnitus treatment option. Promising results have been obtained by consecutive stimulation of lateral frontal and auditory brain regions. We investigated a combined stimulation paradigm targeting the anterior cingulate cortex (ACC) with double cone coil rTMS, followed by stimulation of the temporo-parietal junction area with a figure-of-eight coil. The study was conducted as a randomized, double-blind pilot trial in 40 patients suffering from chronic tinnitus. We compared mediofrontal stimulation with double-cone-coil, (2000 stimuli, 10 Hz) followed by left temporo-parietal stimulation with figure-of-eight-coil (2000 stimuli, 1 Hz) to left dorsolateral-prefrontal-cortex stimulation with figure-of-eight-coil (2000 stimuli, 10 Hz) followed by temporo-parietal stimulation with figure-of-eight-coil (2000 stimuli, 1 Hz). The stimulation was feasible with comparable dropout rates in both study arms; no severe adverse events were registered. Responder rates did not differ in both study arms. There was a significant main effect of time for the change in the TQ score, but no significant time x group interaction. This pilot study demonstrated the feasibility of combined mediofrontal/temporoparietal-rTMS-stimulation with double cone coil in tinnitus patients but failed to show better outcome compared to an actively rTMS treated control group.

*Chronic Tinnitus* Subjective tinnitus is characterized by the perception of sound in the absence of a corresponding sound source^1^. Most people have already experienced an occasional and transient perception of sound in their ears or head^2^. Typically, this perception is reversible and subsides approximately between a few seconds to a few days. However, in about 5 to 15% of the adult general population these phantom sounds are perceived chronically^3^,^4^. Approximately 1% of the general population report severe tinnitus-related impairment of daily living^5^ and seek medical help^6^. No evidence-based established treatments for curing chronic tinnitus or for reduction of its loudness are available^7^,^8^.

## Transcranial magnetic stimulation

Transcranial magnetic stimulation (TMS) is a non-invasive tool for inducing electric currents in the brain^9^. Magnetic fields created by a short and strong electric current circulating within a coil, penetrate the skull painlessly and result in depolarization of superficial cortical neurons^10^. This technique has gained increasing attention as a potential clinical tool for the treatment of a variety of neuropsychiatric disorders (for an overview please see^11^) including chronic tinnitus^12^,^13^,^14^. Most, but not all sham-controlled studies revealed beneficial effects with this approach in the treatment of tinnitus (for the last five years e.g.^15^,^16^,^17^,^18^,^19^,^20^,^21^,^22^,^23^. TMS is regarded a safe technique with only few adverse effects^24^ and has been proven to be feasible for both in- and out-patient treatment^25^.

## Boosting rTMS effects in chronic tinnitus by combined stimulation protocols – the involvement of the limbic system

However, rTMS treatment results are currently burdened by only moderate improvement and high inter-individual variability^12^,^13^,^14^ indicating the need for optimization strategies. As hypothesized already more than 20 years ago^26^ and confirmed by recent neuroimaging findings, tinnitus is related to (i) abnormal activity in both auditory and non-auditory brain regions^27^,^28^,^29^,^30^,^31^,^32^ and to (ii) abnormal functional connectivity between these regions^33^,^34^,^35^,^36^,^37^. Moreover, abnormal functional connectivity has been demonstrated in patients reporting bothersome tinnitus^38^, whereas normal functional connectivity was detected in tinnitus patients without bother^39^. Based on these results combined rTMS stimulation paradigms targeting both auditory and affect regulatory brain regions have been developed and given hopeful results up to now^17^,^40^,^41^.

Typically, the direct effect of the magnetic field produced by an rTMS coil remains limited to superficial brain areas and does not reach subcortical brain structures^42^. However, in the treatment of depression it has been postulated that the rTMS effects created by targeting the dorsolateral prefrontal cortex (DLPFC) rely on the modulation of the anterior cingulate cortex (ACC) via fronto-cingulate functional connectivity^43^. Moreover, the antidepressant response of rTMS over the DLPFC in the treatment of depression depends on baseline activity of the anterior cingulate^44^,^45^,^46^. Since the ACC is critically involved in tinnitus distress as well^47^ it may be hypothesized analogously, that a similar mode of action involving the ACC is the case in the combination treatment of chronic tinnitus targeting both the DLPFC and the auditory cortex^17^,^41^,^48^.

The newly developed double cone coil (DCC) exerts an expanded penetration depth based on its angled geometry. A positron emission tomography study revealed that mediofrontal TMS using a double-cone coil is apt to modulate deeper located brain areas such as the dorsal and subgenual ACC^49^. The ACC appears to be the critical link in the interaction of dorsal/cortical and ventral/limbic networks involved in the experience and regulation of emotion^50^. A subdivision of the ACC, the dorsal ACC (dACC) projects to the prefrontal cortex and plays a critical role in executive functions by influencing multiple cognitive processes^51^ while another subdivision of the ACC, the subgenual ACC (sgACC) has been shown to be involved in emotion experience and processing^52^.

As the ACC has been shown to be critically involved in depression^53^,^54^ **a**nterior-**c**ingulate-cortex-stimulation using a **d**ouble **c**one coil (=ACDC-stimulation) has been investigated as an innovative approach for the treatment of affective disorders very recently and has given promising results so far^55^,^56^.

First clinical experience in tinnitus patients has been gained by Vanneste *et al.* applying double-cone-coil-rTMS to the dorsal frontal cortex of 78 patients^57^. The results indicated that both tinnitus intensity and tinnitus distress reduction following prefrontal double-cone-coil-rTMS were frequency dependent. The same group in Antwerp published a second study reporting differences between a single session and repeated sessions of 1Hz TMS by double-cone coil prefrontal stimulation for the improvement of tinnitus in 73 tinnitus patients receiving single or repetitive session(s) of TMS using a DCC placed over the prefrontal cortex. The results indicated that both single sessions as well as multiple sessions suppress tinnitus distress and tinnitus intensity transiently. It was further shown that multiple sessions of prefrontal double-cone-coil-rTMS generate a higher suppression effect in comparison to a single session and that more patients responded to repeated sessions of 1 Hz stimulation in comparison to a single session^21^.

Based on these previous results and the notion, that, in view of the involvement of both auditory and non-auditory brain regions in tinnitus^27^,^28^,^29^,^30^,^31^,^32^, combined stimulation protocols exert greater effects^17^,^40^,^41^ the aim of the present study was to investigate a combined stimulation paradigm primarily targeting the ACC by mediofrontal stimulation with double-cone-coil followed by conventional stimulation with figure-of-8-coil applied to the temporo-parietal junction area. Foci of interest were the evaluation of safety, feasibility, and clinical effectiveness of a combined anterior-cingulate-cortex-rTMS using a **d**ouble-**c**one-coil in the treatment of patients with chronic **ti**nnitus (TiCDC).

## Methods and Materials

### Study design

**Figure 1:**
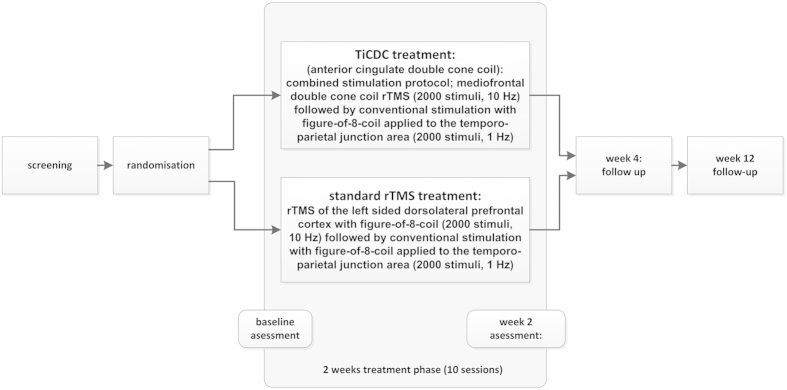
Structure of the present “TiCDC” pilot trial; (for detailed information see methods’ section).

The study was conducted in a randomized, double-blind, parallel-group design with an active control group. Patients were assessed at screening, baseline, end of treatment (week 2), and two follow-up-visits taking place in week 4 and 12. We compared two combined study protocols consisting of medial frontal stimulation with double cone coil (study arm 1) vs. conventional prefrontal left DLPFC-stimulation (study arm 2/control group) both followed by stimulation of the left temporo-parietal junction area. Both study arms consisted of a total of 4000 stimuli per day. Detailed information on the study protocol and the applied stimulation parameters is provided in Fig. 1. The active control condition was chosen as this combined treatment approach to our current knowledge has proven the highest effectiveness compared to other approaches in larger samples up to now^58^.

The study was approved by the Ethics Committee at the University of Regensburg. All participants gave written informed consent after a comprehensive explanation of the procedures. All study methods and procedures were carried out in accordance with the approved guidelines. The study had been previously registered with clinicaltrials.gov (identifier NCT01663311, date of registration: July 23, 2012).

### Patient enrolment

Inclusion criterion was subjective tinnitus with duration of more than six months. Exclusion criteria comprise objective tinnitus (with a treatable cause), start of other treatments for tinnitus 3 months before study enrolment, presence of clinically relevant psychiatric comorbidities or unstable medical conditions, history or evidence of significant brain malformation or neoplasm, history of head injuries, cerebral vascular events, presence of irremovable metal objects in and around body, pregnancy, alcohol abuse or intake of illicit substances, and history of prior TMS treatment.

Patients were recruited for participation in the study after presentation in the outpatient clinic of the Interdisciplinary Tinnitus Centre at the University of Regensburg, Regensburg, Germany.

### Outcome measures

Primary objective was the responder rate at week 2 evaluation (=end of rTMS treatment, see Fig. 1). Clinical response was defined as a minimal reduction of 5 points in the tinnitus questionnaire (TQ)^59^ as compared to baseline according to Adamchic *et al.*^60^. Accordingly, patients with a tinnitus reduction <5 TQ points were defined as “non-responders”. Secondary objectives were the assessment of adverse events and safety information for all available measurement time points as well as changes from baseline to final visit for the TQ, the Tinnitus Handicap Inventory (THI)^61^, the TBF-12-questionnaire scores^62^ (a short version of the THI consisting of 12 selected items), the Major Depression Inventory (MDI)^63^, the Clinical Global Impression (CGI-CHANGE), and quality of life measured by WHOQoL-Bref-questionnaire ratings^64^ where higher scores represent higher quality of life. Data were assessed according to international standards^65^,^66^ and registered in a tinnitus database following ICH-GCP-regulations^67^.

### Study conduct

Participants of both study arms were treated applying combined stimulation paradigms. Conventional rTMS (study arm 2) of the left DLPFC was applied at 10 Hz frequency (2000 stimuli per session, 40 trains with 50 stimuli, intertrain interval 25 seconds) with a figure-of-eight (=butterfly) coil (Cool-B65, Magventure A/S, Denmark) followed by 1 Hz stimulation of the left temporoparietal junction area^68^,^69^ (2000 stimuli per session). TiCDC-stimulation (study arm 1) was performed over the medial frontal cortex at equal frequency using a double cone coil (Cool-D-B50, Magventure A/S, Denmark) followed by stimulation of the left temporo-parietal junction area in the same manner as in study arm 2^68^,^69^. The coils were powered by a MagPro X100 stimulator (Magventure A/S, Denmark). Stimulation was performed at 110% resting motor threshold (RMT) for the butterfly coil applications, at 100% RMT for the double cone coil, or at 60% maximum stimulator output (MSO) when RMT exceeded 54% MSO (mean RMT = 45.8 ± 10.1% MSO). RMT was defined as the lowest intensity sufficient to produce left thenar muscle activation (magnetic evoked potentials >50 mV) with a single pulse delivered to the motor cortex in at least 5 out of 10 trials. For treatment of the left DLPFC the conventional butterfly-coil was positioned 6 cm anterior of the left motor hotspot in sagittal direction^25^, mediofrontal ACDC-stimulation followed the protocol described by Hayward *et al.* positioning the coil 1.5 cm anterior to one third of the distance from the nasion to the inion^49^. Treatment was started on Mondays and patients received a total of 4000 stimuli/day on 10 subsequent working days.

### Statistical analyses

All data are displayed as mean ± standard deviation. In case of missing data the last observation was carried forward, dropout participants were excluded from analysis. Responder rates were compared by chi-square tests of independence. For secondary outcome measures we calculated analyses of variance (ANOVA) with the within-subjects factor time (baseline vs. final visit) and the between-subjects factor group (“TiCDC” vs. “standard rTMS” protocol). CGI scores were compared using chi-square tests. All statistical tests were conducted two-tailed, unadjusted for multiple comparisons due to the pilot study character of the trial, and a value of p < 0.05 was assumed as statistically significant. The sample size was set in a range similar to prior actively controlled pilot trials regarding rTMS-based treatment of chronic tinnitus (e.g.^41^,^70^), and was intended to a) estimate and gain first data regarding adverse effects and tolerability of the treatment, and b) provide orientation about clinical effectiveness of the treatment thus providing the basis for further statistical power calculations. Statistical data analysis was performed with IBM SPSS Statistics for Windows, Version 22.0 (released 2013. IBM Corp., Armonk, NY).

## Results

### Patient population

**Figure 2:**
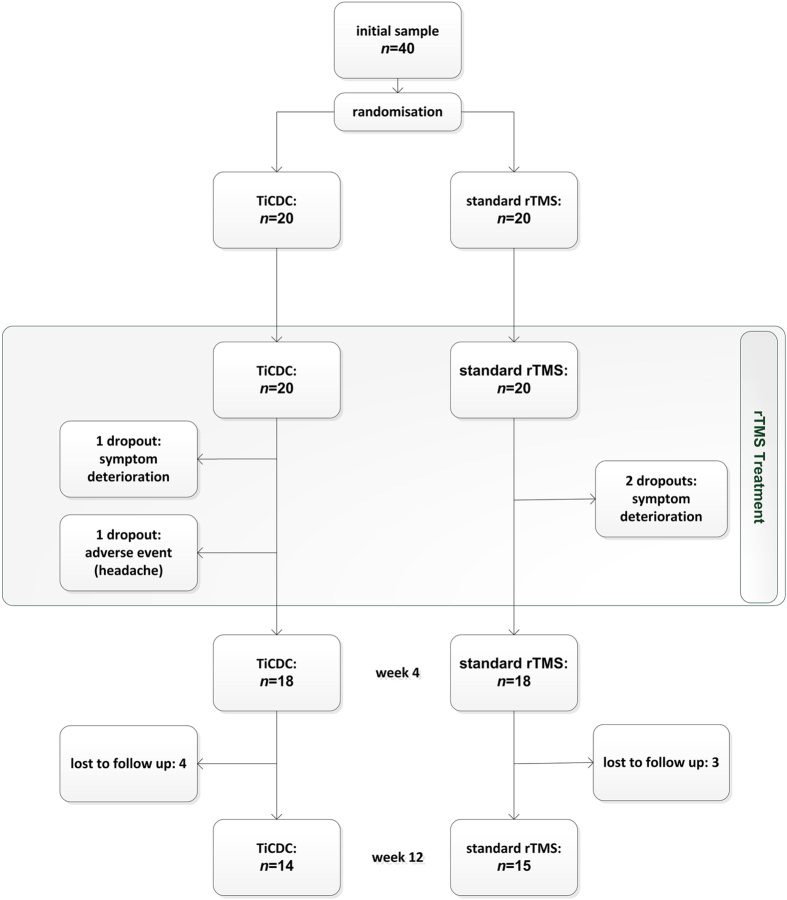
TiCDC study patient flow.

Forty patients were randomized for participation in the study. One participant aborted the stimulation due to headache (TiCDC-participant after session 7). Three study subjects withdrew their consent during the stimulation period due to deterioration of their tinnitus (one TiCDC-participant after session 2; two standard-group-participants after session 1 and session 5). These four subjects were not included in the per-protocol analysis resulting in an analytical sample of 36 patients. The comparison of participants of both treatment groups revealed no differences in the clinical and demographic baseline characteristics. Detailed information is provided in Table 1. Seven patients (standard rTMS: 3; TiCDC: 4) were lost to follow up between week 4 and week 12 (see Fig. 2).

### Safety

No severe adverse events occurred during the course of the study. The incidence of adverse events was comparable in both study arms: six patients complained about headache (3 TiCDC/3 standard rTMS control group/1 dropout (TiCDC)), a deterioration of tinnitus was complained in one TiCDC-participant (leading to the second dropout of TiCDC-group) and two control-group-participants (2 dropouts), dizziness or a deterioration of pre-existing dizziness was reported by 1 patient of each group. Other adverse events included local pain (TiCDC: 1 report) and feelings of ear pressure on both sides (control group: 1 report).

### Primary outcome

As a first step of the data analysis we aimed to compare the number of responders and non-responders in both treatment arms. The primary outcome criterion (responder rate week 2 vs. baseline) turned out non-significant (see Table 2) leading to acceptance of our null-hypothesis (no difference in responder rates comparing both study arms at end of treatment time point). The percentage of responders in the TiCDC arm was 6/18 or 33%, and the percentage of responders in the standard arm was 5/18 or 28%. This represents a difference of 5% with a 95% CI extending from −23% to 33%. For further detailed information see Table 2 (upper part).

### Secondary outcomes

Response rates at week 12 (=end of study) turned out non-significant (χ^2^(1) = 1.870, *p* = 0.171). With regard to the ANOVAs comparing secondary outcomes such as change in TQ, THI, TBF12, MDI, and WHOQoL-questionnaires, the main effect of time turned out significant for the change in the TQ score (baseline score: 33.08 ± 18.26; score at week 12: 29.89 ± 19.11) and marginally significant for the THI with effect sizes being moderate to high (for details see left part of Table 3). No significant changes over time were observed for the remaining outcome measures. The interaction effects between time and group turned out non-significant at uncorrected level (all p’s>.05). For detailed information see right part of Table 3. The observation that the two stimulation protocols did not differ in their clinical efficacy was met by the subjective evaluation of the patients: clinical global impression scales turned out non-significant between groups at week 2 visit and week 12 visit (see Table 4).

**Figure 3:**
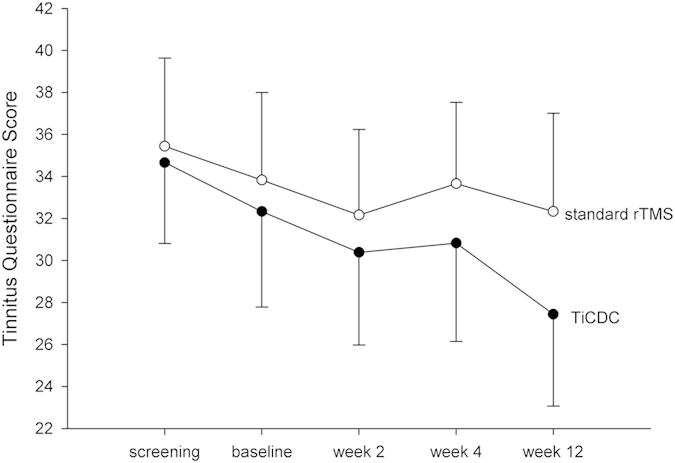
Tinnitus Questionnaire Scores (mean/sem). rTMS treatment took place between baseline and week 2 visit. Week 4 and 12 visits represent follow up.

Graphical information on the TQ score at the different assessment time points is depicted in Fig. 3.

## Discussion

In our pilot study examining 10 sessions of TiCDC-stimulation of the anterior cingulate cortex combined with 1 Hz stimulation of the temporo-parietal junction area this paradigm was well tolerated by the majority of patients, adverse events and dropout rates/reasons were comparable among both study arms and matched prior observations^24^. TiCDC-stimulation turned out to be non-superior to standard rTMS combining butterfly-coil-stimulation of the left DLPFC followed by 1 Hz stimulation of the temporo-parietal junction area regarding both primary and secondary outcome measures.

Notably, the present study was a pilot study, which investigated the stimulation of the anterior cingulated cortex with a double cone coil following suggestions of Vanneste and colleagues^21^,^57^,^71^. An actively controlled parallel group study design has been used to investigate whether this approach is superior to an established protocol of combined frontal and temporal stimulation, which has shown promising results so far^17^,^41^,^48^. Active control conditions have especially been recommended in rTMS studies due to the inherent limitations of sham conditions^72^. For sure, the threshold of demonstrating superiority compared to an established and clinically efficient stimulation protocol was set high. Moreover, the treatment duration of 10 sessions was chosen for feasibility reasons, even if earlier studies especially regarding the treatment of depression have shown better results with an extended treatment duration up to 6 weeks^73^,^74^. Thus, in the interpretation of results the limited power of the study with its relatively small sample size of 2×20 participants, the contrast to an actively treated control group, and the short treatment duration of only 10 sessions rTMS have to be taken into account.

### Pathophysiological considerations concerning the ACC’s role in chronic tinnitus

The anterior cingulate cortex is known to be critically involved in the attentional control of sensory processing^75^,^76^,^77^,^78^. In detail, top-down inhibitory mechanisms that originate in the prefrontal lobe have been shown to play an important role in auditory processing^79^ and in the generation of tinnitus^80^. Thus, it is tempting to speculate, that rTMS-mediated modulation of the ACC might strengthen deficient inhibitory top-down mechanisms in tinnitus patients. According to the model of chronic tinnitus originating from overlapping, concurrently-activated and interacting neural networks containing auditory, emotion regulatory, attention-processing and memory networks^36^ the ACC plays a central role in several of these functional networks. In detail, it has been proposed, that high-frequency, gamma band, synchronized neural activity in the sensory cortex representing the auditory hyperactivity in tinnitus patients becomes a conscious percept when connected to larger coactivated “(self-)awareness” and “salience” brain networks comprising the anterior cingulate cortex and the bilateral insula^81^. If tinnitus is stressful, this is reflected by a simultaneously co-activated nonspecific distress network consisting of again the anterior cingulate cortex, anterior insula, and amygdala^36^. Memory mechanisms play a role in the persistence of the awareness of the phantom percept, as well as in the reinforcement of the associated distress^36^. As part of the historical Papez circuit, the anterior cingulate cortex is critically involved in such memory processing exerting its connections to other brain areas such as the parahippocampal region that has been extinsively investigated regarding the pathophysiology of chronic tinnitus up to now^82^,^83^. Moreover, the ACC has also been shown to be involved in default mode network activity^84^,^85^, a fact, that – especially in consideration of reduced focality due to enhanced penetration depth in double cone coil rTMS – might have further distracted the results reported in this study.

### Prior studies regarding double cone coil TMS

To our knowledge this is the first randomized and actively controlled clinical study applying double cone coil TMS in patients suffering from chronic tinnitus. A pubmed search with the term “double cone” AND “TMS” retrieved 26 hits (access date: 02.12.2014). Vanneste and colleagues reported the successful treatment of a patient suffering from a medication-resistant chronic depression by 10 sessions of ACDC-stimulation applying the same stimulation parameters as used in our pilot study (mediofrontal stimulation with double cone coil at 10 Hz, 2000 stimuli/session)^55^. We added 2000 stimuli of 1 Hz-rTMS to the temporo-parietal junction area^68^,^69^ applying a figure-of-8-coil transforming this treatment approach into a combined rTMS setting. The patient treated by Vanneste and colleagues was – although showing a relatively high RMT of 65% MSO – treated with a stimulation intensity of only 40% MSO because higher amplitudes were not well tolerated in this case. The Beck Depression Inventory score improved by 27%, and the two subscales of the Hospital Anxiety Depression Scale (HADS), namely depression (40%) and anxiety (33%) improved as well. Along with the clinical improvement source-localized electrophysiological resting state activity changed in the dACC and sgACC in this patient in comparison to a normative group^55^. Two large case series reporting stimulation of an almost identical target as in the first stimulation component of the TiCDC-protocol, the so-called dorsomedial prefrontal cortex (dmPFC) applying the same stimulation device, were very recently published by Salomons *et al.*^86^ and Downar *et al.*^87^. The former investigated whether resting-state functional connectivity predicted response to dmPFC-rTMS treatment in depressive patients. In twenty-five individuals with treatment-refractory MDD higher baseline cortico-cortical connectivity and lower cortico-thalamic, cortico-striatal, and cortico-limbic connectivity were associated with better treatment outcomes^86^. Downar and colleagues^87^ reported results of 20 rTMS sessions to the dorsomedial prefrontal cortex in 47 patients with a medication-resistant major depressive episode. Responders and non-responders showed opposite patterns of hemispheric lateralization in the connectivity of dorsomedial and dorsolateral regions to this same ventromedial region^87^. Very recently, the authors of the present study have published the results of a three-armed, randomized, double-blind clinical study applying ACDC-stimulation in a sample of inpatients suffering from major depression^56^. Forty-five patients suffering a moderate to severe depressive episode were randomized to receive 15 sessions of either conventional rTMS of the left DLPFC (“butterfly-rTMS”; 10 Hz; 2000 stimuli/day, RMT 110%), mediofrontal double cone coil stimulation of the anterior cingulate cortex (“ACDC-rTMS” with equal parameters), or sham-stimulation. There was a significant group x time interaction effect regarding the change in the 21-items Hamilton Rating Scale for Depression (HAMD) from baseline to the end of treatment, which served as primary outcome criterion. Post-hoc testing revealed a significant effect for the comparison ACDC vs. butterfly at week 3 (end of treatment). Equalling our present experience, no severe adverse events had occurred during the study, ACDC-stimulation was well tolerated by the majority of patients similar like butterfly-rTMS and sham-stimulation.

### Work to do

Despite encouraging results of previous applications of mediofrontal double-cone-coil-rTMS in patients suffering from depression, the present study failed to confirm the alternative hypothesis of superiority as part of a combined treatment setting in subjects suffering from chronic tinnitus. This might be due to limited power of the present study, which would require comparisons to placebo-controlled study groups in larger samples. Apart from that, it is very likely that our understanding of stimulation settings is yet too mechanistic and in addition to the effect in the stimulated cortical area the impact of the stimulation on network activity and connectivity has to be taken into account in more detail^88^. Previous studies have already demonstrated that rTMS with a butterfly coil on the left DLPFC modulates the blood flow in the ACC^89^ via functional connectivity of the DLPFC to deeper located mood-regulatory brain structures. It remains to be investigated by further studies whether stimulation of the DLPFC with the butterfly-coil and dorsomedial double–cone-coil stimulation have a differential effect on ACC activity. The lack of a difference between the two protocols in our study could be either due to a similar effect of both protocols on the ACC or to the non-relevance of a potential differential effect for tinnitus treatment. Therefore, it is vital for a detailed understanding of stimulation sites and parameter optimization to systematically assess this network connectivity in tinnitus patients applying different techniques such as fMRI and source-localized EEG during stimulation. Moreover, these approaches could enable a deeper insight into the pathophysiological underpinnings of direct ACC modulation by double-cone-coil-rTMS taking into account the current uncertainty of optimum stimulation parameters.

However, despite these limitations of the present study and the negative results our data provide novel insights in the feasibility of double-cone-coil rTMS targeting the ACC in patients suffering from chronic tinnitus. Moreover, they allocate an estimation of clinical effects, thus allowing for further methodological adjustments and enabling power calculations for upcoming studies. In summary our pilot data confirm the potential of TiCDC-stimulation as a non-invasive, safe and well tolerated method of brain stimulation in the treatment of chronic tinnitus, but failed to demonstrate superiority compared to the clinical effectiveness of a combinatory rTMS study group addressing both the left DLPFC and the temporo-parietal junction area.

## Additional Information

**How to cite this article**: Kreuzer, P. M. *et al.* Combined rTMS treatment targeting the Anterior Cingulate and the Temporal Cortex for the Treatment of Chronic Tinnitus. *Sci. Rep.* **5**, 18028; doi: 10.1038/srep18028 (2015).

## Figures and Tables

**Table 1:** Baseline characteristics of patients.

	TiCDC(*n* = 18)	Standard rTMS(*n* = 18)	Statistics
gender (female/male)	5/13	6/12	χ^2^(1) = 0.131,*p* = 0.717
age (years)	50.9 ± 10.7	54.4 ± 9.0	*t*(34) = −1.054,*p* = 0.299
tinnitus duration in months	78.3 ± 79.7	97.0 ± 143.8	*t*(34) = −0.483,*p* = 0.632
tinnitus laterality (right/left/both)	1/2/15	2/5/11	χ^2^(5) = 3.562,*p* = 0.614
TQ total score	32.3 ± 19.3	33.8 ± 17.7	*t*(34) = −0.243,*p* = 0.809
THI total score	38.3 ± 21.8	36.6 ± 21.8	*t*(34) = 0.245,*p* = 0.808
MDI	7.1 ± 5.8	6.9 ± 6.3	*t*(34) = 0.110,*p* = 0.913

TQ: Tinnitus Questionnaire, THI: Tinnitus Handicap Inventory, MDI: Major Depression Inventory.

**Table 2:** Responder Analysis.

Week 2 visit (end of treatment)- primary outcome -	responder	non-responder
both groups	11	25
TiCDC	6	12
standard rTMS	5	13
statistics	χ^2^(1) = 0.131, *p* = 0.717	
Week 12 visit (end of study)		
both groups	14	22
TiCDC	9	9
standard rTMS	5	13
statistics	χ^2^(1) = 1.870; *p* = 0.171	

**Table 3:** Secondary outcomes: ANOVA with between subjects factor group (‘TiCDC’ vs. ‘standard rTMS’) and within subjects factor time (baseline vs. week 12).

	main effect time			interaction effects for group x time (baseline vs. week 12)		
	*F* (1, 34)	*p*-value	part. Eta^2^	*F* (1, 34)	*p*-value	part. Eta^2^
TQ	5.085	0.031	0.130	1.431	0.240	0.040
THI	3.459	0.072	0.092	3.459	0.072	0.092
TBF12	0.150	0.701	0.004	2.265	0.142	0.062
MDI	2.222	0.145	0.061	1.502	0.229	0.042
WHOQOL1	1.956	0.171	0.054	3.412	0.073	0.091
WHOQOL2	0.176	0.678	0.005	1.477	0.233	0.042
WHOQOL3	0.103	0.751	0.003	1.499	0.229	0.042
WHOQOL4	0.067	0.789	0.002	1.351	0.253	0.038

TQ: Tinnitus Questionnaire, THI: Tinnitus Handicap Inventory, MDI: Major Depression Inventory, WHOQoL: World Health Organization Quality of Life Questionnaire (Domain 1–4), TBF12: Tinnitus Impairment Questionnaire (short version of the THI consisting of 12 items).

**Table 4:** Clinical Global Impression (CGI) at week 2 and week 12.

Week 2 visit(end of treatment)	much better	minimallybetter	no change	minimallyworse
both groups	3	8	16	6
TiCDC	2	4	7	3
standard rTMS	1	4	9	3
statistics	χ^2^(3, *N* = 33) = 0.554, *p* = 0.907			
Week 12 visit (end of study)				
both groups	3	5	17	4
TiCDC	1	2	9	2
standard rTMS	2	3	8	2
statistics	χ^2^(3, *N* = 29) = 0.558; *p* = 0.906			
